# The Minneapolis Meeting

**Published:** 1885-07

**Authors:** 


### THE MINNEAPOLIS MEETING.

By reference to another page of this number, it will be seen that the Committee of Arrangements are planning for a royal time in connection with the meeting of the American Dental Association. The time is convenient and the opportunity an unusual one for a pleasurable vacation. The attractions are so well set forth in the notice from the Chairman of the Committee, that it is needless to say more. But it is hoped that there will be a large attendance at the meeting. The pleasure of the journey thither will be much enhanced if the dentists can go on the same trains. Therefore, we venture to suggest an itinerary. If delegates from New York will leave that city by the 6.00 P. M. train of Sunday, they will be in Buffalo at 6.10 Monday morning, in time to take the Michigan Central "Flyer," which will land them in Chicago at 9.00 P. M. of the same day, and they will arrive at Minneapolis at 3.10 P. M. Tuesday. Or, if they leave New York at 9.15 P. M. of Saturday, they will arrive in Minneapolis at 7.15 on Monday evening. Delegates from Boston will be obliged to leave on Saturday evening, to arrive in time for this meeting. The members from New York can go as far as Buffalo by either the West Shore or the New York Central Roads, but from that point the Michigan Central presents special attractions. It gives its passengers the finest opportunity to see Niagara Falls that is presented from any one point, without leaving the train. They will also have the benefit of the famous dining cars of this road, and will be in Chicago in time to join the delegates from other points who will meet them.

To avoid all worry, to be able to arrive in Minneapolis in sufficient time to get a good night's rest before the commencement of the meeting, that the delegates may not be completely jaded and fagged out at the very outset, we take the liberty to suggest that the members leave New York Saturday evening, and Buffalo Sunday noon, reaching Niagara at 1.20 P. M., Detroit 8.45 P. M., Chicago 8.00 A. M., arriving in Minneapolis at 7.15 Monday evening. Thus the journey of 1400 miles will be agreeably broken by occasional brief stops, and the delegates will arrive in time for a preliminary rest.

